# PKCδ-dependent signaling mediates ethambutol-induced toxic effects on human retinal pigment cells

**Published:** 2011-06-09

**Authors:** Rong Kung Tsai, Ming Shan He, Zih-Yao Chen, Wen Chen Wu, Wen Sheng Wu

**Affiliations:** 1 Department of Ophthalmology, Buddhist Tzu Chi General Hospital, Hualien, Taiwan; 2 Department of Ophthalmology and Visual Science, Hualien, Taiwan; 3 Department of Medical Technology, Tzu Chi University, Hualien, Taiwan; 4 Department of Ophthalmology, Kaohsiung Medical University, Kaohsiung, Taiwan

## Abstract

**Purpose:**

Our previous report demonstrated that ethambutol (EMB) might induce cytoplasmic vacuolization and reduce the uptake of photoreceptor rod outer segments (ROS) in retinal pigment epithelium (RPE) cells, which are mediated via a protein kinase C (PKC)-dependent pathway. In the present study, we sought to identify the PKC isozyme(s) involved.

**Methods:**

EMB-induced cytoplasmic vacuolization and uptake of ROS were observed under a phase contrast microscope. Western blots were performed to observe the membrane translocation of PKC isozymes and cytoplasmic release of cathepsin D. Quantitative PCR were performed to analyze gene expression of PKCδ. Human RPE cell line RPE50 and ARPE19 cells were pretreated with specific inhibitors or transfected with shRNAs of various PKC isozymes, including PKCα, β, ε, γ, and δ, to examine whether EMB-induced toxic effects were prevented.

**Results:**

In RPE50 cells, gene expression of PKCδ on both mRNA and protein levels was induced by EMB within 30 min to 3 h. EMB-induced cytoplasmic vacuolization in both RPE50 and ARPE19 cells was prevented by pretreating the cells with a specific inhibitor of PKCδ, Rottlerin, or depletion of PKCδ by shRNA. EMB-triggered reduction of ROS uptake was also significantly suppressed by pretreatment with Rottlerin, or depletion of PKCδ by shRNA technology. In contrast, pretreatment of the cells with specific inhibitors of PKCα, β, ε, or γ, or depletion of PKCα or β didn’t influence the aforementioned EMB-triggered toxic effects. In addition, in RPE50, EMB induced the release of lysosomal enzyme cathepsin D into cytosol within 30 min to 6 h, which was also prevented by Rottlerin.

**Conclusions:**

EMB-induced vacuole formation, cytoplasmic release of cathepsin D, and reduction of phagocytosis in RPE are intimately correlated and regulated by the PKCδ signal pathway.

## Introduction

Ethambutol (EMB) is routinely used as an anti-mycobacterial agent, especially in the treatment of tuberculosis. However, EMB can cause vision impairment, ethambutol-induced optic neuropathy (EON), in 1%–5% of patients [1]. Some patients have suffered irreversible vision loss [2,3].

It has been suggested that the cause of EON might be associated with disturbance of the optic nerve that is induced by EMB via an excitotoxicity pathway [4-9]. However, the toxic effects of EMB on retinal cells were also highlighted in recent studies [10-13]. For example, one clinical study that used multifocal electroretinography (mfERG) to examine EON patients suggested that the visual dysfunction might be entirely attributable to toxicity of the retina rather than optic nerve [11]. Another study demonstrated an obvious retinal abnormality in EON patients, including retinal pigment epithelial change, macular edema, and flame-shaped hemorrhages consistent with abnormal ERG findings [13]. Moreover, it was reported that 55.6% (15/27) of patients with EON had an abnormal Arden ratio in electrooculography (EOG) examinations, which indicated that EMB can cause retinal pigment epithelial (RPE) cell dysfunction [14]. In the retina, the RPE is located between the choroid capillary layer and the light-sensitive outer segments of the photoreceptors, and is supposed to be the area most susceptible to EMB-induced pathological effects. Indeed, our recent studies have demonstrated that EMB may induce toxic effects such as cytosolic vacuolization and reduced phagocytic activity in human RPE-derived cells, including RPE50 and ARPE19 [12]. We also found that protein kinase C (PKC) activity can be induced by EMB and is required for EMB-induced vacuolar formation; however, the PKC isozyme(s) responsible for the EMB-induced toxic effects remain(s) unidentified.

Thus far, at least 12 isoforms of tissue-specific PKC have been found and can be divided into three major groups: the classic PKCs (cPKC: PKCα, PKCβI, PKCβII, and PKCγ), the novel PKCs (nPKC: PKCδ, PKCε, PKCη, PKCθ), and the atypical PKCs (aPKC: PKCζ, PKCλ, and PKCι) [15,16]. Ten of the PKC isozymes are present in cultured human RPE cells [17]. Among them, PKCα, PKC βII, PKCδ, and PKCε have been reported to be associated with pathological effects of RPE [18]. In the present study, we sought to identify which PKC isozyme is responsible for the toxic effects of EMB on RPE.

## Methods

### Human RPE cell line

RPE50 is a primary culture of human RPE cells provided by the Tissue Culture Center, New York Eye and Ear Infirmary. This cell line was isolated from an anonymous donor sample not referable to any patient [19]. RPE50 has been used for studying the effects of oxidative stress on ion channels [20] and also for cell cycle analysis and gene expression [21]. ARPE19, purchased from the Bioresource Collection and Research Center (BCRC, Hsinchu, Taiwan) is more differentiated than RPE50, having been characterized by ZO-1 and RPE65, two differentiation markers of RPE, in our previous study [12]. Both cell lines were maintained in a 1:1 mixture of Dulbecco’s Modified Eagle’s Medium (DMEM) and a F12 medium containing 1.2 g/l sodium bicarbonate, 2.5 mM L-glutamine, 15 mM 4-(2-hydroxyethyl)-1-piperazine-ethanesulfonic acid (HEPES), 0.5 mM sodium pyruvate, and 10% fetal bovine serum (FBS).

### Chemicals and antibodies

Ethambutol dihydro-chloride (EMB), TCPK-trypsin, and soybean trypsin inhibitor were purchased from Sigma (St. Louis, MO). Various PKC isozyme inhibitors, including Rottlerin, Go6976, Ro32–0432, RBX, bisindolymaleimide, DAPH-7, and HBDDE were purchased from Calbiochem (La Jolla, CA). Antibodies against PKCα, β, γ, δ and cathepsin D were purchased from Santa Cruz Biotechnology (Santa Cruz, CA).

### Fractionation of the cellular extract

#### The cytosolic and membrane fraction

Briefly, the cells were suspended in a hypotonic buffer (10 mM Tris in pH 7.4, 50 mM NaCl, 0.3 mM Na-orthovanadate, 50 mM NaF, 1 mM DTT, 10 g/ml leupeptin, and 5 ug/ml aprotinin) and incubated at 4 °C for 30 min. The cell suspensions were homogenized and centrifuged at 594 ×g for 3 min. The supernatants were then subjected to ultracentrifugation at 22190 ×g for 1 h, and the resulting supernatants obtained provided the cytosolic fraction. The pellets were then dissolved in a lysis buffer containing 50 mM Tris, 50 mM NaCl, 1% Triton X-100, 0.1% SDS, 0.3 mM Na-orthovanadate, 50 mM NaF, 1 mM DDT, 10 μg/ml leupeptin, and 5 μg/ml aprotinin. Following a second centrifugation at 22190× g for 1 h, the supernatants obtained provided the membrane fraction.

#### The lysosomal fraction

The crude lysosomal fraction was prepared according to the protocol of a Lysosme Isolation Kit (Sigma) but with some modification. Briefly, the harvested cells were broken by freezing and thawing in the lysis buffer at −80 °C, followed by centrifugation at 1,000× g for 10 min. The pellet from the cell fragmentation was discarded and the supernatant was centrifuged again at 20,000× g for 20 min. The pellet was then re-suspended in a lysis buffer for crude lysosomal fraction (CLF) containing a mixture of mitochondria, lysosomes, peroxisomes, and endoplasmic reticulum.

### Immunoblotting

Immunoblotting was performed using a standard procedure. Briefly, the cells were lysed and equal amounts of protein in each sample were separated by 12% SDS/PAGE, and then transferred to PVDF membranes. Membranes were blocked in 5% dry milk and were probed with antibodies against molecules of interest. Following incubation with an alkaline phosphatase-conjugated secondary antibody, proteins were visualized with NBT/BCIP for color development. For quantization, the intensity of each specific band was estimated using gel digitizing software, UN-SCAN-IT gel version 5.1.

### Quantitative RT/PCR

Cellular mRNA was purified with an Ultraspec kit (Biotech, Houston, TX) followed by cDNA synthesis using Reverse-it^TM^ (ABgene, Surrey, UK). Real-time PCR of PKCδ was performed using a QuantiTect SYBR PCR kit (Qiagen, Crawley, UK) in an ABI 7300 real-time PCR system (Applied Biosystems, Foster City, CA).

PCR mixtures were preincubated at 95 °C for 15 min to activate the polymerase (Qiagen, Valencia, CA). Each of the 40 PCR cycles consisted of 16 s of denaturation at 94 °C, annealing of primers for 30 s at 55 °C, and 15 s of extension at 72 °C. The relative amounts of mRNA were calculated by the 7300 system SDS Software using glyceraldehyde 3-phosphate dehydrogenase (*GAPDH*) as the internal control.

The primer sequences used for *PKCδ* and *GAPDH* were: 5′-GAA GCA GGG ATT AAA GTG TG-3′ and reverse: 5′-TTC TTC TCG AAA CCC TGA TA-3′ (192 bp). *GAPDH* forward: 5′-CGG AGT CAA CGG ATT TGG TCG TAT-3′ and reverse: 5′-AGC CTT CTC CAT GGT GGT GAA GAC-3′ (301 bp).

### Observation and quantitation of cytoplasmic vacuolization

**Figure 1:**
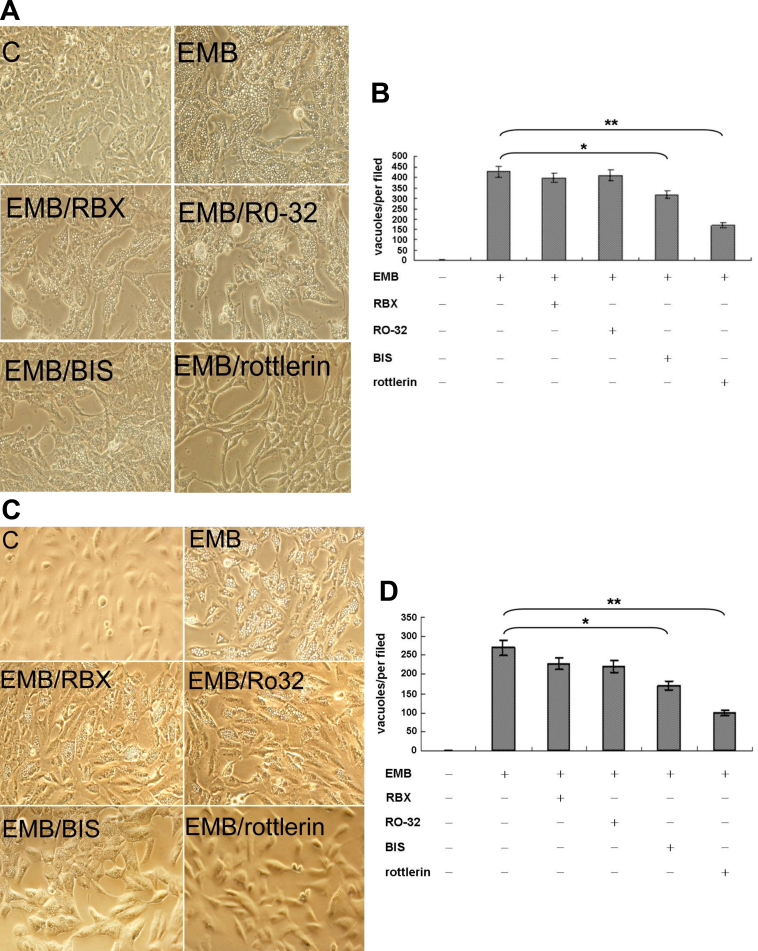
Protein kinase C (PKC)δ was required for ethambutol (EMB)-induced vacuolar formation in retinal pigment epithelium (RPE). RPE50 (**A**) or ARPE19 (**C**) cells were untreated (denoted as C), treated with 8.0 mM EMB alone, or EMB plus the indicated inhibitors of PKC isozymes for 24 h. Pictures were taken under a phase contrast microscope with 200× magnification. This result is representative of three reproducible experiments. (**B**) and (**D**) are quantitations of cytoplasmic vacuolization for (**A**) and (**C**), respectively, performed as described in Methods. The number of vacuoles/per field were compared between indicated treatment groups using one way ANOVA followed by Dunnett’s post hoc tests. The asterisks (* and **) indicate statistical significance (p<0.05 and p<0.005, respectively, n=3) between the indicated groups.

**Figure 2:**
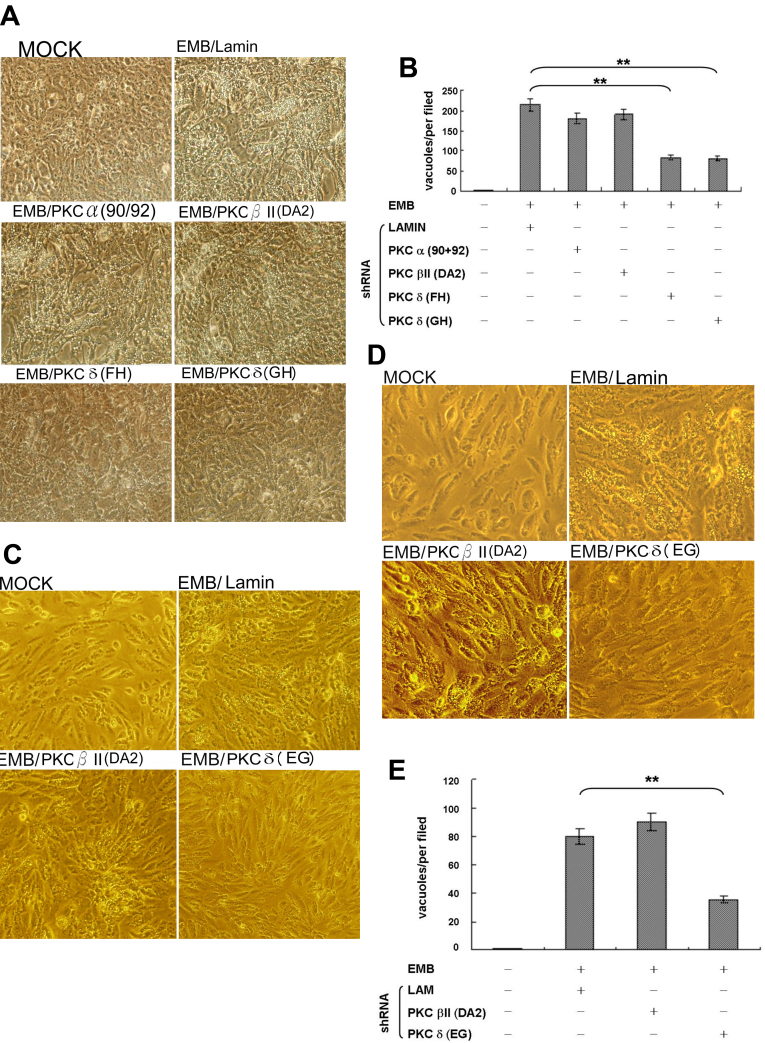
Depletion of protein kinase C (PKC)δ prevented ethambutol (EMB)-induced vacuolar formation. Retinal pigment epithelium (RPE)50 (**A**) or ARPE19 (**C**) and (**D**) were transfected with none (MOCK), shRNA of lamin (control shRNA), or combinations of shRNA fragments of PKCα (90/92 in A), PKC βII (D/A2 in **A** and **C**), or PKCδ (F/H, G/H in A and E/G in C), for 36 h followed by treatment with 0.8 mM EMB for 24 h. Pictures were taken under a phase contrast microscope with 200× (**A**, **C**) and 400× (**D**) magnification. (**B**) and (**E**) are quantitations of cytoplasmic vacuolization for (**A**) and (**D**), respectively, performed as described in Methods. These results are representative of five reproducible experiments. The number of vacuoles/per field were compared between indicated treatment groups using one way ANOVA followed by Dunnett’s post hoc tests. The asterisks (* and **) indicate statistical significance (p<0.05 and p<0.005, respectively, n=5) between the indicated groups.

For comparison of the extent of cytoplasmic vacuolization exhibited in RPE cells under various treatments (Figure 1 and Figure 2), the number of vacuoles within 90%–100% of confluent cells under the 200× magnification field of a phase contrast microscope were counted. Eight fields per well (of a 24-well plate) were scored and averaged (data not shown). The average number of vacuoles/per field were compared between indicated treatment groups using one way ANOVA followed by Dunnett’s post hoc test. Each experiment was repeated and validated by two other investigators, both of whom were unaware of the treatment groups before microscopic observation.

### Photoreceptor rod outer segments (ROS) preparation

ROS were prepared according to previous reports but with modifications [22]. Briefly, retinas from 10 fresh pig eyes obtained from a local slaughterhouse were prepared under dim red light. The retinas were dissected and placed in a homogenizing solution (20% W/V sucrose, 20 mM Tris acetate (pH 7.2), 2 mM MgCl_2_, 10 mM glucose, and 5 mM taurine), followed by filtration through cheesecloth. The filtrates were layered on 25%–60% W/V continuous sucrose gradients containing 20 mM Tris acetate (pH 7.2), 10 mM glucose, and 5 mM taurine, and were then centrifuged at 22190× g for 45 min at 4 °C. A single pink band in the upper third of the gradient was collected, diluted with 0.02 M Tris buffer (PH7.2) by fivefold, and centrifuged at 2375× g for 10 min. The pellets were resuspended in 1 ml of 0.02 M Tris buffer (pH 7.2) and digested with TCPK-trypsin at 23 °C for 15 min. The reaction was then stopped by the addition of 10 μl of soybean trypsin inhibitor (4 mg/ml). The trypsin-treated ROS were washed with 10 ml of 0.02 M Tris buffer (pH 7.2) containing 10% sucrose by centrifugation at 7988× g for 10 min. The ROS pellets were resuspended at a concentration of approximately 4 mg/ml in 0.02 M sodium phosphate (pH 7.2) containing 10% W/V sucrose and were stored at −80 °C.

### In vitro phagocytosis assay

Phagocytosis of ROS by RPE was performed according to previous reports [23]. The ROS were labeled with 1 mg/ml of fluorescein isothiocyanate (FITC, Molecular Probes) in 0.1 M NaCO_3_ (pH 9.0), for 1 h in the dark, followed by washing and re-suspension in the cell culture medium with 2.5% sucrose at a concentration of 5×10^7^ ROS/ml. The labeled ROS (2×10^6^) were incubated with RPE cells in 50 μl of the culture medium containing 2.5% sucrose. Phagocytosis was allowed to occur for the experimental times indicated. At the end of each time period, the cells were incubated with 0.2% trypan blue in PBS containing 1 mM MgCl_2_ and 0.2 mM CaCl_2_ (PBS-CM) for 10 min to quench the FITC fluorescence derived from the externally bound particles [24]. The cells were then fixed by ice-cold methanol for 5 min, followed by 3% paraformaldehyde in PBS-CM for 10 min at room temperature. Finally, nuclei were stained with 1 μg/ml propidium iodide (PI) in PBS-CM for 20 min; samples were then mounted for observation under a fluorescent microscope (200× magnification; Olympus, Tokyo, Japan). For quantization, the number of ROS and cells were counted separately. The phagocytic activity (ROS uptake) was calculated as the average of the number of ROS/cell in 8 randomly selected fields. The relative phagocytic activity in each experimental group was defined as the value for ROS uptake divided by those of the control RPE.

### shRNA technology for depletion of PKC isozymes

Lentiviral plasmids encoding shRNAs targeting different regions of the indicated mRNA were obtained from RNAi Core Laboratory in Academia Sinica, Taiwan. Cells at 60% confluence were transfected with 0.26 ng/μl effective shRNAs, either alone or in combination, using Effectene transfection reagent (Invitrogen Ltd, Renfrew, UK) according to the manufacturer's protocol. The required treatments were performed after the cells were transiently transfected with shRNAs for 36 h. The target sequence of each of the shRNA fragments for *PKCα*, *PKCβII*, and *PKCδ* used in this study are listed in Table 1.

**Table 1:** The target sequence of shRNA fragments for PKCα, βII, and δ

**PKC isozyme**	**Fragment name**	**Targeting sequence**
PKCα	90	CTTTGGAGTTTCGGAGCTGAT
	92	CATGGAACTCAGGCAGAAATT
PKCβ	D	CTATCCCAAGTCTATGTCCAA
	A2	GCTGAAAGAATCGGACAAAGA
PKCδ	E	GGCCGCTTTGAACTCTACCGT
	F	CAAGGCTACAAATGCAGGCAA
	G	GCAAGACAACAGTGGGACCTA
	H	GCAGGGATTAAAGTGTGAAGA

### Statistical analysis

**Figure 3:**
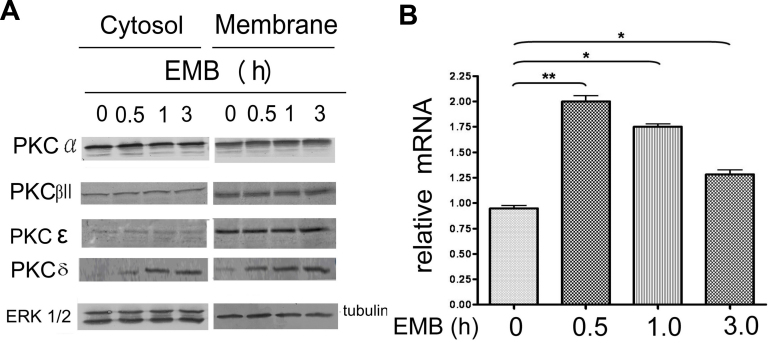
Ethambutol (EMB) did not induce membrane translocation of various protein kinase C (PKC) isozymes but did induce gene expression of PKCδ in retinal pigment epithelium (RPE). RPE50 cells were treated with 8.0 mM EMB for 0, 0.5, 1 and 3 h. Western blots of cytosolic and membrane PKCα, βII, and δ (**A**), and real time RT/PCR (**B**) of PKCδ were performed. In (**A**), ERK and tubulin were included as internal controls for cytosolic and membrane proteins, respectively. The results are representative of four repeated experiments. In (**B**), relative amounts of PKCδ mRNA for each sample were calculated, taking the sample of time zero as 1.0. The asterisks (* and **) indicate statistical significance (p<0.05 and p<0.005, respectively, n=3) between the indicated groups.

**Figure 4:**
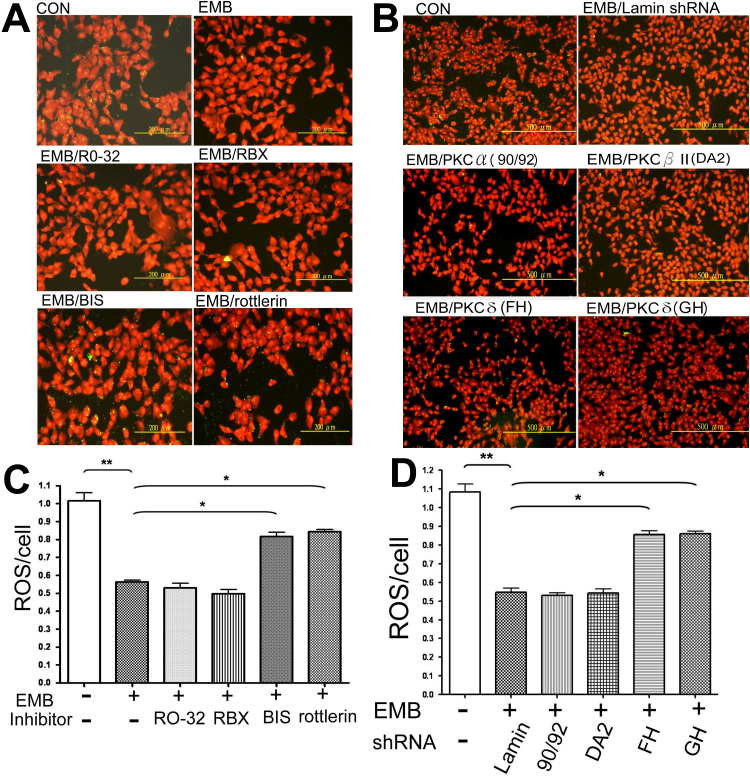
Inhibition or depletion of protein kinase C (PKCδ) prevented ethambutol (EMB)-induced suppression of the uptake of rod outer segments (ROS) in RPE50 cells. The phagocytosis assay was performed in RPE50. In (**A**) the fluorescein isothiocyanate (FITC)-labeled ROS were added to the cells in the absence (con) or presence of EMB alone, or EMB plus various PKC isozymes (as indicated) for 2.5 h. In (**B**), the cells were untransfected (con), transfected with shRNA of control (lamin), or various PKC isozymes for 36 h, followed by the addition of the FITC-labeled ROS added in the absence (CON) or presence of EMB for 2.5 h. The FITC-labeled ROS (green dot) up-take by the cells was observed under a phase contrast microscope (200× magnification). The nuclei were stained with PI (red) as counterstaining. The figures shown are representative of six to eight repeated experiments. (**C**) and (**D**) exhibit quantitative data for (**A**) and (**B**), respectively. The extent of ROS uptake was expressed as ROS/cell obtained from the average of the number of ROS and the cells in eight fields, under a phase contrast microscope. The asterisks (* and **) indicate statistical significance (p<0.05 and p<0.005, respectively, n=7) between the indicated groups.

Paired Student's *t* tests were conducted to statistically analyze quantitative RT/PCR (Figure 3) and relative ROS uptake (Figure 4C,D) between the indicated groups. All quantitative studies were performed at least in triplicate, with the results expressed as the mean±SD as appropriate. The preventive effects of various inhibitors or the shRNA of PKC isozymes on EMB-induced cytoplasmic vacuolization (Figure 1 and Figure 2) were evaluated by one-way ANOVA followed by Dunnett’s post hoc comparisons. Differences were considered to be significant at p<0.05. One or two asterisks (* and **) indicate statistical significance (p<0.05 and p<0.005, respectively) between groups.

## Results

### PKCδ activity was required for EMB-induced vacuolar formation in RPE

**Figure 5:**
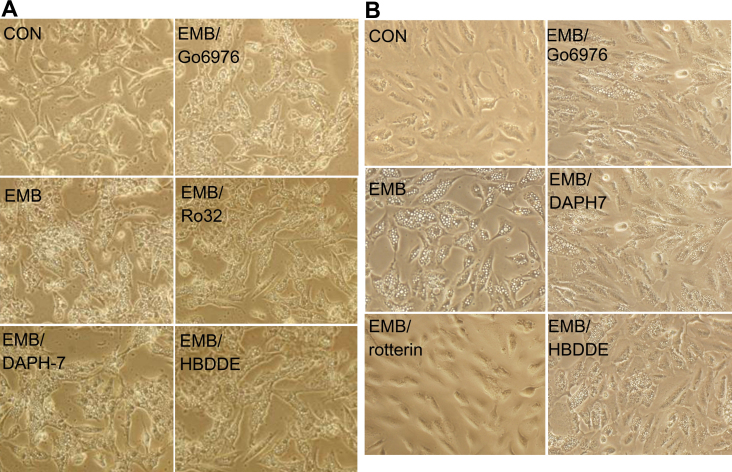
Inhibitors of protein kinase C (PKC) isozymes other than PKC δ could not prevent ethambutol (EMB)-induced vacuolar formation in retinal pigment epithelium (RPE). RPE50 (**A**) and ARPE19 (**B**) cells were untreated (CON), treated with 8 mM EMB, or EMB plus various PKC isozymes as indicated, for 24 h. Pictures were taken under a phase contrast microscope with 200× magnification. This result is representative of three reproducible experiments.

Previously, we have demonstrated that EMB could induce PKC-dependent cytotoxic effects such as cytoplasmic vacuolization in two human RPE cell lines, RPE50 and ARPE19. We further screened for the PKC isozyme(s) responsible for the EMB-induced vacuolization, using various pharmacological inhibitors of PKC isozymes, including PKCα, β, γ, δ, and ε (Table 2). RPE50 cells were pretreated with 2 μM of Rottlerin (for PKCδ, IC_50_=2–6 μM), 5 nM Go6976 (for PKCα, β, and γ, IC_50_=2–10 nM), 28 nM Ro32–0432 (for PKCα and βI, IC_50_=9 nM, 28 nM), 2 μM RBX (PKCβII, IC_50_=1–2 μM), 5uM bisindolymaleimide (BIS; for PKCα, δ, ζ, η, and ε, IC_50_=1–5 μM), 500 nM DAPH-7, (for PKCβII and βI, IC_50_=410 nM, 3.8 μM) or 50 μM HBDDE (for PKCα and γ, IC_50_=43 μM, 50 μM) to observe whether EMB-triggered vacuolization in RPE can be prevented. As demonstrated in Figure 1A,B, the vacuolar formation induced by EMB was very prominent at 24 h and was suppressed by 61% in RPE50 cells pre-treated with 2 μM of Rottlerin (p<0.005, ANOVA). Pretreatment of RPE50 with BIS exhibited partially suppressive effects on vacuolar formation (by 26%; p<0.05, ANOVA), and the vacuoles were smaller than those observed in the “EMB-treated only” group. In contrast, EMB-induced vacuolization could not be significantly prevented in RPE50 pre-treated with inhibitors of other PKC isozymes, including Go6976, Ro32–0432, RBX, DAPH-7, and HBDDE (Figure 1A,B and Figure 5A). Similar results were obtained using the more differentiated RPE, ARPE19, as the target cells. As demonstrated in Figure 1C,D, pretreatment of ARPE19 with 2 μM of Rottlerin abolished EMB-induced vacuolization by 91% (p<0.005, ANOVA) whereas pretreatment of ARPE19 with BIS resulted in partial suppression of EMB-induced vacuolization (by 37%; p<0.05, ANOVA) and formation of smaller vacuoles as compared with that induced by EMB. In contrast, Go6976, Ro32–0432, RBX, DAPH-7, or HBDDE didn’t exhibit a significant preventive effect on EMB-induced vacuolization (Figure 1C,D and Figure 5B). Taken together, these results suggested that PKCδ activity was specifically required for EMB-triggered vacuolization in both RPE50 and ARPE 19.

**Table 2:** Various PKC isozyme inhibitors

**Inhibitors**	**PKC isozyme**	IC_50_*
Bisindolymaleimide	(PKC α, δ, ζ, η, ε)	2–5 μM
Go6976	(PKCα.β.γ)	2–10 nM
Rottlerin	(PKCδ)	3–6 μM
HBDDE	(PKCα.γ)	43 μM, 50 μM
DAPH-7	(PKCβII.βI)	410 nM, 3.8 μM
Ro32–0432	(PKCα. βI)	9 nM, 28 nM
RBX	(PKC βII)	1–2 μM

*IC_50_: concentration for 50% inhibition.

### EMB induced gene expression of PKCδ in RPE50

**Figure 6:**
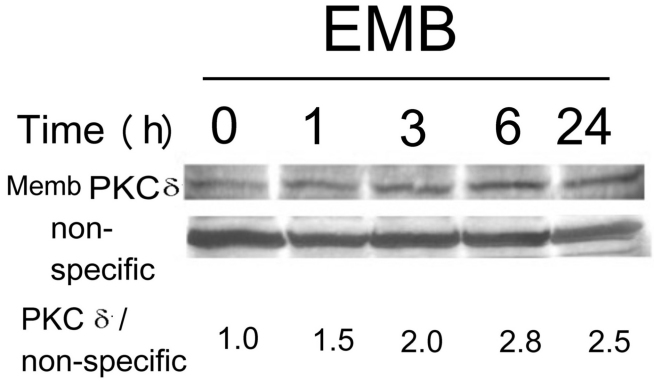
Extended time-course analysis of ethambutol (EMB)-induced elevation of membrane protein kinase C (PKCδ). RPE50 cells were treated with 8.0 mM EMB for 0, 1, 3, 6, and 24 h. A western blot of membrane PKC δ was performed. A non-specific band in the Ponceau S stained blot was included as an internal control. The result is representative of two repeated experiments. The relative ratios of the band intensity of PKC δ versus a non-specific band, taking the ratio of time zero as 1.0, are shown below.

We then investigated which PKC isozyme(s) could be activated by EMB by observing whether any of them exhibited “cytosol to membrane translocation,” which is indicative of PKC activation in various cell types, including RPE cells [25]. As demonstrated in western blots for both the cytosolic and membrane fraction of RPE50 cells, neither a decrease in cytosolic or an increase in membrane fraction of PKCα, β-II, or ε was observed after treatment with EMB within 1 to 3 h (Figure 3A). Surprisingly, PKCδ significantly increased in both cytosolic and membrane fractions after treatment of EMB for 30 min and showed a 3–4 fold increase within 1 to 3 h (Figure 3A). Extended time-course analysis demonstrated that the EMB-induced elevation of membrane PKCδ was still observed at 6 h and was sustained until 24 h (Figure 6). These results implied that the gene expression of PKCδ in RPE50 was upregulated by EMB. Indeed, PKCδ mRNA showed a twofold elevation after treatment with EMB for 30 min and this declined to a 1.75 and 1.25 fold elevation at 1 and 3 h, respectively, as shown by real time RT/PCR (Figure 3B).

### Transfection of shRNA of *PKCδ* but not of other PKC isozymes prevented EMB-induced vacuolar formation in RPE50 and ARPE19

**Figure 7:**
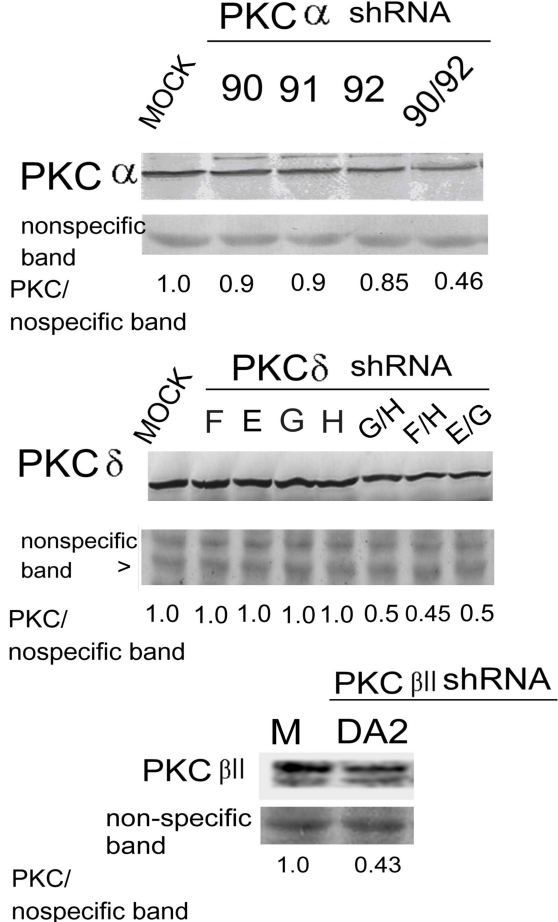
Depletion of protein kinase C (PKCα) and PKCδ by shRNA. RPE50 cells were transfected with none (MOCK), or various shRNAs fragments (either alone or in combination) of indicated PKC isozymes. Western blots of PKCα (upper panel), PKCδ (middle panel), or PKCβII (lower panel) were performed using non-specific bands in the Ponceau S stained blots as internal controls. The relative ratios of the band intensity of each PKC versus a nonspecific band, taking the ratio of MOCK as 1.0, are shown below. The results were the average of three repeated experiments with a C.V. of 6.0–8.0.

To this stage in the study, it appeared that PKCδ was the only PKC isozyme induced by EMB to mediate cytoplasmic vacuolization. We further investigated whether blocking of PKCδ expression could prevent EMB-induced vacuolization in RPE50. Various small hairpin RNA (shRNA) fragments of *PKCδ*, *PKCα*, and *PKCβII* targeting different regions on each mRNA, either alone or in combination (with two fragments), were screened for their efficiency to knock down each isozyme. As demonstrated in the western blot (Figure 7), transient transfection of RPE50 cells with combined shRNA fragments of *PKCα* (90 plus 92), *PKCβII* (D plus A2), or *PKCδ* (F plus H, G plus H or G plus E) for 36 h could effectively depress the expression of each PKC isozyme by between 50%–65% as compared with those in the MOCK-transfected cells. However, single shRNA fragments of *PKCα* and *PKCδ* did not exhibit significant depletion efficiency. Subsequently, the effective shRNA combinations were transiently transfected into RPE50 for 36 h and this was followed by EMB treatment. As demonstrated in Figure 2A,B, the EMB-induced vacuolization in RPE50 at 24 h was dramatically suppressed in the cells transfected with *PKCδ* shRNA (F plus H or G plus H), by between 60%–65% as compared with that of RPE50 transfected with control (lamin) shRNA (p<0.005, ANOVA). In contrast, transfection of the cells with shRNA of *PKCα* (90 plus 92) and *PKCβII* (D plus A2) did not show significant preventive effects. Similar results were obtained using ARPE19. As demonstrated in Figure 2C,E, the EMB-induced vacuolization in ARPE19 at 24 h was suppressed by 57% in the cells transfected with shRNA (E plus G) of *PKCδ* but not *PKCβII* (D plus A2), as compared with those observed in cells transfected with control (lamin) shRNA (p<0.05, ANOVA). The magnification of Figure 2C was increased and is provided in Figure 2D, demonstrating a clearer observation of the vacuoles. The efficiency of *PKCδ* shRNA (F plus H or G plus H) for blocking EMB-induced vacuolization was not so prominent in ARPE19 (data not shown) as it was for those observed in RPE50 and is probably due to different cell contexts.

### PKCδ inhibitors and shRNA rescued the EMB-induced reduction of ROS uptake in RPE50 and ARPE19

**Figure 8:**
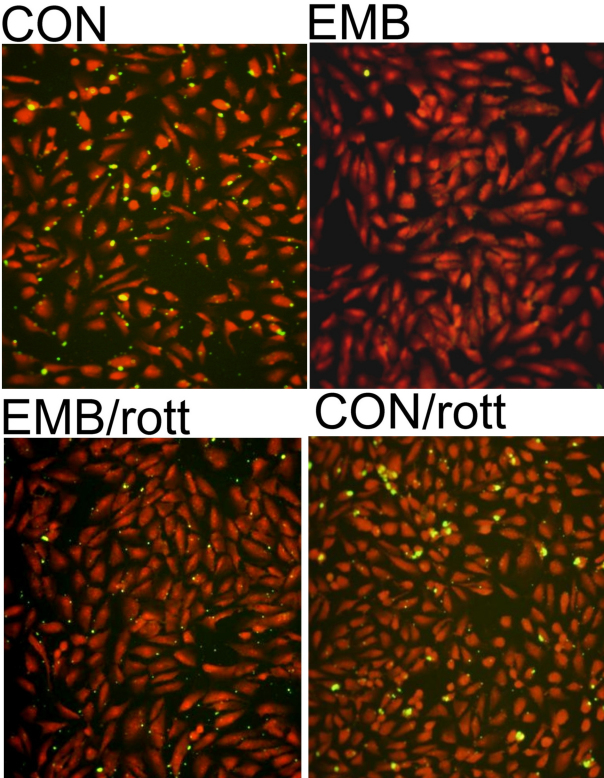
Inhibition of protein kinase C (PKCδ) prevented ethambutol (EMB)-induced suppression of rod outer segments (ROS) uptake in ARPE 19. The fluorescein isothiocyanate (FITC)-labeled ROS were added to the cells without any treatment (CON), in the presence of 8 mM EMB alone, EMB plus 3 μM Rottlerin (EMB/rott), or Rottlerin alone (CON/rott) in ARPE19 for 5 h. The FITC-labeled ROS (green dot) uptake by the cells were observed under a phase contrast microscope (200× magnification). The nuclei were stained with PI (red) as counterstaining. The figure shown was representative of two repeated experiments.

Previously, we found that EMB may decrease the phagocytic activity of RPE50 as revealed by a reduction in the uptake of rod outer segments (ROS) after treatment with EMB for 2.5 to 5 h [12]. Thus, we further investigated whether this effect was also mediated by *PKCδ*. As demonstrated in Figure 4A,B, treatment of RPE50 with EMB for 2.5 h suppressed the ability of RPE to uptake ROS by 44%. Pre-treatment of the cells with BIS and Rottlerin (but not inhibitors of the other PKC isozymes) significantly precluded the EMB-triggered reduction of ROS uptake by 36 and 40%, respectively (Figure 4A,B). Furthermore, suppression of ROS uptake by EMB was attenuated by 28%–30% in RPE50 transfected with *PKCδ* shRNA (GH and FH), as compared with that in cells transfected with control (lamin) shRNA (Figure 4C,D). In contrast, transfection of the cells with shRNA of the other PKC isozymes (90 plus 92, and D plus A2 for *PKCα* and *PKCβII*, respectively) did not show any preventive effects. Similar results were obtained using ARPE19. As demonstrated in Figure 8, treatment of EMB for 5.0 h dramatically suppressed ROS uptake in ARPE19. A cotreatment with 2 μM Rottlerin greatly prevented EMB-induced reduction of ROS uptake. As a negative control, 2 μM Rottlerin did not influence ROS uptake in the absence of EMB (CON/rott). These results indicated that *PKCδ* was specifically required for EMB-induced suppression of ROS uptake in RPE.

### EMB-induced elevation of cytosolic cathepsin D in a PKCδ-dependent manner

**Figure 9:**
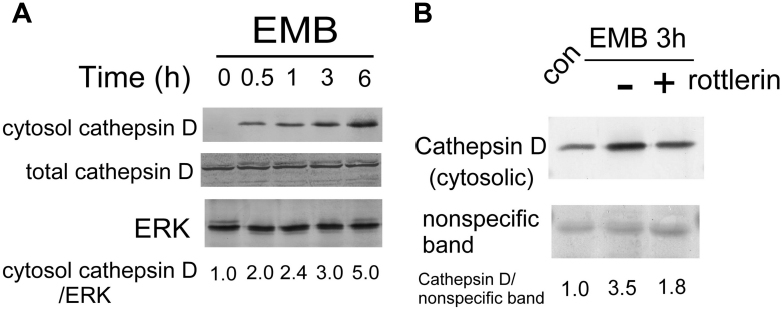
Ethambutol (EMB) induced cytosolic release of cathepsin D in a protein kinase C (PKCδ)-dependent manner in RPE 50. RPE50 cells were untreated (con; **B**) or treated with 0.8 mM EMB for 0, 0.5, 1, 3, and 6 h (**A**) or 3 h with or without pretreatment of 2 μM Rottlerin (**B**). Western blots of total cathepsin D (**A**) or cytosolic cathepsin D (**A**, **B**) were performed using ERK (**A**) or a nonspecific band in the Ponceau S stained blot (**B**) as an internal control. The relative ratios of the band intensity of cathepsin D versus ERK (**A**) or the nonspecific band (**B**), taking the ratio of time zero (**A**) or untreated (**B**) as 1.0, are shown below. The results were an average of three repeated experiments with a C.V. of 6.0–8.0.

**Figure 10:**
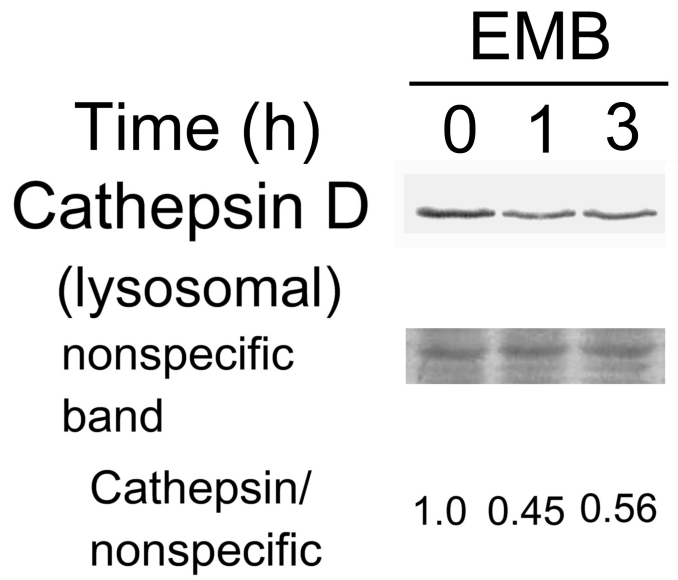
Ethambutol (EMB) induced release of cathepsin D from lysosome in RPE50 cells. RPE50 cells were treated with 0.8 mM EMB for 0, 1, and 3 h. A western blot of cathepsin D in the crude lysosomal fraction was performed. A nonspecific band in the Ponceau S stained blot was included as an internal control. The relative ratios of the band intensity of cathepsin D versus the nonspecific band, taking the ratio at time zero as 1.0, are shown . The results were an average of two repeated experiments with a coefficient of variation (CV) of 6.0%–8.0%.

One intriguing issue with regards to EMB-induced vacuolization was addressed by a recent report suggesting that the vacuoles were derived from lysosomes [10]. The report demonstrated that EMB might induce lysosomal membrane permeabilization (LMP) resulting in leakage of lysosomal content and subsequent vacuolar formation. One of the most abundant lysosome components released into cytosol was the acid phosphatase cathepsin D [10]. Thus, we further examined whether EMB may induce cytoplasmic release of cathepsin D and whether PKCδ is involved. As demonstrated in Figure 9A, cathepsin D can not be detected in cytosol, in contrast with the high basal level of cathepsin D in the total lysate of untreated RPE50. After treatment with EMB for 30 min, cytosolic cathepsin D was increased 2.0-fold and showed a further 2.4, 3.0, and 5.0-fold increase at 1, 3, and 6 h, respectively, while total cathepsin D was not influenced. However, cathepsin D was detected in the crude lysosomal fraction of RPE50, which was gradually decreased by 40% after treatment of EMB for 1–3 h (Figure 10). Interestingly, pretreatment of RPE50 with 2 μM Rottlerin suppressed the EMB-induced cytosolic elevation of cathepsin D by 49% (p<0.05, ANOVA) at 3 h (Figure 9B), implicating that this phenomenon was also mediated by PKCδ.

## Discussion

### The role of RPE in EMB-induced adverse effects on retinas

In this report, we identified the critical signal molecule responsible for the toxic effects of EMB on RPE, which is probably one of the most critical target cells involved in EMB-induced retinopathy. Physiologically, RPE plays important roles in the maintenance of the blood-retinal barrier and in the transportation of trophic factors and nutrients to the retina. Moreover, RPE closely interacts with photoreceptors to maintain visual function. Specifically, RPE may serve as a phagocyte for the uptake and catabolism of the daily shed rod outer segments to recycle the visual pigments and maintain homeostasis of photoreceptors [26,27]. Thus, long-term administration of EMB may affect certain important functions of RPE, such as phagocytic activity, leading to retinopathy. This speculation was substantiated in our previous in vitro and in vivo study [12].

### Various EMB-induced toxic effects in RPE were closely related

Our results demonstrated that EMB-induced cytosolic vacuolization and reduction of phagocytosis in both RPE50 and ARPE19 were mediated by PKCδ (Figure 1, Figure 2, and Figure 4). This strongly implies a close relationship between the two phenotypic changes. In a recent study, the vacuoles induced by EMB were demonstrated to be derived from lysosomes with increased membrane permeabilization (LMP), as revealed by the release of lysosomal components such as cathepsin D into cytosol [10]. On the other hand, lysosomes are responsible for degrading the phagocytosed photoreceptor outer segment disks in RPE via a phagosome–lysosomal fusion [28]. Taken together, it is tempting to speculate that EMB may trigger lysosomal dysfunction and the subsequent reduction in the phagocytic ability of RPE. Interestingly, our results demonstrated that EMB might also induce the cytosolic release of lysosomal cathepsin D in RPE50 in a PKCδ-dependent manner (Figure 9B). This finding further strengthens the notion that EMB-induced cytosolic vacuolization, LMP, and the reduction of phagocytosis are closely related cellular events regulated by the same signal pathway.

### Role of PKCδ in mediating pathological processes in RPE

We found that PKCδ was the only PKC isozyme responsible for EMB-induced toxic effects in RPE. Unlike PKCβ (which stimulates growth) and PKCε (which acts as an oncogene and promotes tumors in nude mice), PKCδ generally slows proliferation, induces cell cycle arrest, enhances the differentiation of various cell lines [29-31], and is intimately associated with DNA damage-induced apoptosis [32]. In addition, PKCδ plays an important role in several pathological processes, including vascular complications associated with diabetes [33]. Moreover, PKCδ inhibitors may attenuate reperfusion injury and may improve thrombolysis outcomes [34].

Although EMB did not induce membrane translocation of PKCδ (Figure 3A), EMB-induced gene expression of *PKCδ* was observed at both the protein (Figure 3A) and RNA (Figure 3B) level. Importantly, depletion of *PKCδ* expression prevented EMB-induced toxic effects, including vacuolar formation and reduction of ROS uptake (Figure 2 and Figure 4, respectively). A previous study also demonstrated that activation of *PKCδ* can be achieved by elevating gene expression. For example, p21 (RAS), through its downstream effector PI3K, induces *PKCδ* expression and PKCδ activity through Akt, which is required for cell survival [35].

In summary, we conclude that EMB induced toxic effects on RPE in a PKCδ-dependent manner. In the future, it may be possible to develop pharmacological inhibitors against PKCδ to prevent the adverse effects exerted by EMB on the retina.
